# Differential Pathways to Depressive Symptoms in Students With and Without Special Educational Needs: The Role of Emotional Reactivity and Parent–Teacher Relationships

**DOI:** 10.3390/bs16101830

**Published:** 2026-10-07

**Authors:** Andriani A. Papadaki, Antonios I. Christou, Elias Kourkoutas, Ariadni Argatzopoulou

**Affiliations:** 1 Department of Primary Education, University of Crete, 74100 Rethimno, Greece; ptdep90121@edc.uoc.gr (A.A.P.); eliaskourk@uoc.gr (E.K.); 2 Department of Special Education, University of Thessaly, 38221 Volos, Greece; aargatzop@uth.gr

**Keywords:** social and emotional learning, emotion regulation, emotional reactivity, parent–teacher relationships, perceived acceptance–rejection, inclusive education, special educational needs (SEN), depressive symptoms

## Abstract

Social and emotional competence (SEC) is a core target of school-based social and emotional learning (SEL), yet learner variability may alter how relational experiences and emotion-regulation difficulties are associated with mental-health outcomes. Depressive symptoms were treated as clinically meaningful indicators of psychological distress and, within an SEL-oriented framework, as downstream correlates of difficulties in emotional self-management; they were not treated as interchangeable with low SEC. Participants were 465 Greek students aged 10–14 years (160 in the combined SEN group—94 with documented status and 66 identified by teacher screening—and 305 without SEN) and 51 teachers from 26 schools. Students completed measures of emotional reactivity, perceived parental and teacher acceptance–rejection, and depressive symptoms. Cluster-adjusted path models, bootstrap tests of indirect effects, covariate-adjusted sensitivity analyses, and formal multigroup equality constraints were used. In students without SEN, paternal rejection showed a direct association with depressive symptoms (β = 0.15), whereas maternal rejection showed indirect associations through Recovery (standardized β = 0.011) and Impairment (standardized β = 0.022). In the combined SEN group, teacher rejection (β = 0.18) and emotional impairment (β = 0.38) were directly associated with depressive symptoms, and paternal rejection showed indirect statistical associations through emotional reactivity. In an analysis restricted to the 94 students with documented SEN, the teacher-rejection and Impairment associations persisted, whereas the paternal rejection–Recovery estimate was similar but less statistically certain. Formal multigroup tests supported differences only for maternal rejection—Recovery, maternal rejection—Impairment, and teacher rejection—depressive symptoms; other between-group contrasts were descriptive or inconclusive. The findings illustrate how relational and regulatory correlates may vary across heterogeneous learner groups, without establishing causal or temporal mechanisms. The teacher-rejection finding warrants further study in students with documented SEN, while the paternal pathways require particular caution because the Recovery association was less statistically certain in the smaller documented-only analysis.

## 1. Introduction

Social and emotional learning (SEL) frameworks emphasize the development of social and emotional competence (SEC) as a foundational prerequisite for students’ academic engagement, psychological well-being, and long-term adjustment. Within this perspective, resilience-related processes—particularly those governing emotion regulation and relational functioning—are increasingly recognized as central mechanisms through which learners access, engage with, and benefit from SEL-relevant supports. However, accumulating evidence suggests that these processes do not operate uniformly across students. Rather, learner variability in emotional reactivity and relational sensitivity may result in differentiated pathways to social–emotional outcomes, particularly for students with special educational needs (SEN). Resilience has been extensively studied as a multidimensional construct encompassing psychological, emotional, and behavioral capacities of individuals and systems. Broadly, resilience has been conceptualized as the capacity of a dynamic system to withstand or recover from significant challenges that threaten its stability, viability, or development, as well as the ability to overcome the adverse effects of stress and adversity (Masten, 2001; Ungar, 2011). Over time, the field has produced a wide range of definitions, reflecting the complexity and contextual embeddedness of resilience processes. To address this conceptual heterogeneity, Luthar et al. (2000) distinguished between resilience as a dynamic developmental process and resiliency as a set of individual characteristics that are learned and refined across development.

Despite its theoretical importance, the assessment of resilience has been challenged by the lack of definitional consensus, the interactive nature of individual–context transactions, and difficulties in capturing subtle mechanisms through standardized measurement approaches (Thorne & Kohut, 2007). In response to these challenges, Thorne and Kohut (2007) proposed a three-factor model of personal resiliency designed to facilitate practical application and empirical assessment. This model conceptualizes personal resiliency as comprising: (a) Sense of Relatedness, reflecting competence in forming and maintaining supportive relationships that buffer stress; (b) Sense of Mastery, encompassing optimism, self-efficacy, and adaptability; and (c) Emotional Reactivity, referring to individual differences in sensitivity to emotional stimuli and regulatory capacity in response to stress and adversity.

Within this framework, Emotional Reactivity represents a core component of social and emotional competence, capturing the mechanisms through which environmental demands are translated into internal emotional experiences and behavioral responses. Emotional Reactivity is operationalized across three interrelated dimensions: Sensitivity, Recovery, and Impairment (Thorne & Kohut, 2007). Sensitivity refers to the threshold and intensity of emotional responses, reflecting how quickly and strongly individuals react to emotionally salient stimuli. Recovery captures the duration required to return to baseline functioning following emotional arousal, a process closely linked to vulnerability for internalizing difficulties (Davidson, 2000). Impairment reflects the extent to which emotional arousal disrupts cognitive, behavioral, and social functioning, including difficulties with concentration, behavioral control, and decision-making (Davidson, 2000). Together, these dimensions provide a developmentally sensitive index of emotion regulation and self-management, central domains within SEL frameworks. Consistent with neurocognitive and psychophysiological accounts of emotion development within parent–child dyads, these regulatory capacities emerge and are calibrated through ongoing relational exchanges rather than developing in isolation (Christou & Bacopoulou, 2025).

Social and emotional competence does not develop in isolation but is shaped within relational contexts, particularly interactions with parents and teachers. Interpersonal Perceptions of Acceptance–Rejection Theory (IPARTheory), formerly known as Parental Acceptance–Rejection Theory, provides a robust framework for understanding how perceived relational climates influence children’s emotional and psychological adjustment (Rohner, 1986, 2004). Within IPARTheory, acceptance refers to the expression of warmth, affection, care, and support by significant others, conveyed through both physical (e.g., affection, positive nonverbal cues) and verbal behaviors (e.g., praise, encouragement). In contrast, rejection reflects the withdrawal or absence of warmth and support and may manifest through coldness and lack of affection, hostility and aggression, indifference or neglect, or undifferentiated rejection. Higher scores on acceptance–rejection measures indicate greater perceived rejection and less supportive relational environments.

Relational experiences characterized by rejection have been consistently linked to difficulties in emotion regulation, relational competence, and psychological adjustment. Within educational contexts, teachers function as salient attachment-related figures, and students’ perceptions of teacher acceptance or rejection play a critical role in shaping emotional security, engagement, and well-being. From an SEL perspective, parents and teachers constitute the primary relational infrastructure through which emotion regulation skills, self-management strategies, and social competencies are modeled, scaffolded, and reinforced.

Difficulties in emotional regulation and relational functioning are closely associated with depressive symptomatology during childhood and adolescence. Depressive symptoms encompass affective, cognitive, and behavioral features, including persistent sadness, negative self-evaluations, social withdrawal, attentional difficulties, and disruptions in sleep and appetite (American Psychiatric Association [APA], 2013). Importantly, within SEL-oriented frameworks, depressive symptoms can be conceptualized not solely as indicators of psychopathology but as downstream markers of sustained challenges in self-management, emotional regulation, and relational competence. Such difficulties are particularly consequential during school-age development, given their association with both internalizing and externalizing problems and their tendency to remain under-recognized and under-treated in youth populations (Cicchetti & Toth, 1998; Coyle et al., 2003). This conceptual framing does not treat depressive symptoms and low social–emotional competence as interchangeable constructs; rather, elevated depressive symptoms retain their established clinical significance as a marker of psychological distress while also functioning, within an SEL-oriented lens, as one observable indicator among several of accumulated difficulty in emotional self-management and relational functioning.

Learner variability in these social–emotional processes is especially pronounced among students with special educational needs. In Greece, the term Special Educational Needs (SEN) encompasses a broad spectrum of learning differences, including cognitive, emotional, developmental, and socio-behavioral difficulties that affect learning and school adjustment. SEN may arise from formally diagnosed conditions or from functionally evident challenges that have not yet been formally identified. Students with SEN who attend inclusive educational settings often present with difficulties related to attention, learning, speech and language, emotional regulation, or social behavior, as well as broader social, emotional, and behavioral (SEB) challenges influenced by family and environmental contexts. Because these difficulties differ substantially in etiology, developmental course, and support needs, students grouped under the composite SEN category in the present study may present markedly different relational and regulatory profiles; the grouping therefore captures shared functional vulnerability in inclusive classrooms rather than a diagnostically homogeneous population, a constraint addressed further in the Limitations. In the present study, SEN is conceptualized as an indicator of learner variability in social–emotional functioning rather than as a categorical deficit, consistent with contemporary inclusive education and SEL perspectives. This operationalization aligns with Greek educational legislation (Law 3699/2008), which defines SEN as learning difficulties arising from sensory, cognitive, emotional, social, or developmental factors that significantly affect school adaptation and learning processes (Tsakiridou & Polyzopoulou, 2014). In this population, perceived paternal acceptance–rejection has also been linked to self-perception and peer victimization, underscoring the relational vulnerabilities that can accompany SEN status (Christou et al., 2026c).

Taken together, these frameworks underscore the importance of examining how emotional reactivity and perceived relational contexts jointly shape social–emotional competence outcomes across diverse learner profiles. Understanding whether and how these pathways differ for students with and without SEN is essential for advancing inclusive SEL research and informing the design of educational practices and policies that attend to learner variability rather than assuming uniform responsiveness to relational and emotional supports. A related study drawing on the same sample and data-collection procedure (Papadaki et al., 2026) examined a different resiliency dimension—Sense of Relatedness (Trust, Support, Social Comfort, and Tolerance of Differences)—as a mediator linking perceived parent–teacher relationships to depressive symptoms in students with and without SEN, and reported a pathway structure in which paternal rejection was central for students without SEN. The present study is conceptually and analytically distinct: it examines Emotional Reactivity—a separate RSCA subscale indexing sensitivity, recovery, and functional impairment rather than relational competence—as the regulatory mechanism, allowing a test of whether a fundamentally different component of personal resiliency yields convergent or divergent relational pathways in the same population. As reported in the Section 3, the two studies in fact diverge in which parent’s rejection is most closely tied to depressive symptoms among students without SEN, underscoring that the choice of resiliency mechanism, and not merely the relational predictors, shapes the pathways identified.

Specifically, both studies used the same 465 participants and included perceived parental and teacher rejection and depressive symptoms. The earlier analysis examined Sense of Relatedness as the intervening resiliency dimension, whereas the present analysis examines the distinct Emotional Reactivity dimensions of Sensitivity, Recovery, and Impairment and reports component-specific indirect effects and formal multigroup comparisons.

The present study builds on this body of work by examining emotional reactivity as a potential explanatory pathway linking perceived parental and teacher relationship quality with depressive symptoms in school-aged children and early adolescents. Using a school-based design incorporating student self-report and teacher-informed SEN classification, the study examines these associations separately for students with and without SEN, operationalized as an indicator of learner variability in inclusive classrooms. By distinguishing sensitivity, recovery, and emotion-related functional impairment, the study moves beyond global associations while avoiding the assumption that these dimensions constitute established causal mechanisms. The analysis therefore provides process-oriented, cross-sectional evidence relevant to inclusive SEL frameworks while explicitly recognizing heterogeneity within the broad SEN category.

Based on this framework, the study addressed the following research questions: (1) Do students with and without SEN differ in perceived parental and teacher acceptance–rejection, emotional reactivity, and depressive symptoms? (2) Are perceived maternal, paternal, and teacher rejection directly and indirectly associated with depressive symptoms through emotional reactivity (sensitivity, recovery, and impairment), and do these pathways differ between students with and without SEN? Consistent with attachment and IPARTheory perspectives, we expected that perceived rejection from parents and teachers would be positively associated with emotional reactivity and, in turn, with depressive symptoms. Given the emphasis in classical attachment frameworks on the primacy of the maternal bond, we tentatively expected maternal rejection to show the strongest associations with emotional reactivity and depressive symptoms among students without SEN; however, because students with SEN often depend more heavily on structured, non-parental relational supports at school, we expected that teacher rejection would play a comparatively larger role in this group. We did not have a strong a priori basis for hypothesizing the relative contribution of paternal rejection in either group and treated this as an open empirical question.

## 2. Materials and Methods

### 2.1. Participants

The study used the following reproducible sampling frame. Thirty-two eligible public schools in the prefectures of Heraklion, Chania, and Rethymnon were stratified by educational level (Grades 5–6 versus Grades 7–8) and urban/rural location; 26 schools agreed to participate (school participation rate = 81.3%). Within participating schools, intact classrooms were randomly selected. Of 498 eligible students, 465 provided usable data (student participation rate = 93.4%); 18 were absent during data collection, 9 declined participation, and 6 returned questionnaires with more than 20% missing responses. Students ranged from 10 to 14 years. SEN status was determined using two non-overlapping routes: 94 students had KEDASY-documented diagnoses and inclusion-support records, and 66 additional students (41.3% of the combined SEN group) met the TEACH screening rule without documented SEN status. The latter were identified by teacher-rated functional difficulties, not by an independent SEN diagnosis; the internalizing screening domain includes anxious/depressed and withdrawn/depressed items that overlap conceptually with the depressive-symptom outcome. The screening rule required a score at or above the 75th percentile on either the TEACH internalizing domain (score ≥ 14) or externalizing domain (score ≥ 18). Teachers completed a standardized 60 min briefing and written scoring guide before rating students; 10% of classifications were independently checked by a second trained researcher, yielding agreement of κ = 0.86. Of the total sample, 160 students (34.4%) were classified as having SEN and 305 as not having SEN. The SEN group included 93 boys (58.1%) and differed from the non-SEN group in sex composition (*p* < 0.001). Most students lived in urban areas (95.9%) and attended primary education (n = 353; 75.9%). A total of 51 teachers from 26 schools provided TEACH ratings, assessing an average of 9.1 students each (median = 5.5; range = 3–26).

### 2.2. Measures

Parental Acceptance–Rejection Questionnaire (PARQ; Short Form). Perceived parental relationship quality was assessed using the Child Parental Acceptance–Rejection Questionnaire (PARQ; Short Form) developed by Rohner and colleagues (Rohner et al., 2005) and adapted for use in Greek populations (Giovazolias et al., 2010; Tsaousis et al., 2012). The short form consists of 24 self-report items, administered separately for mothers and fathers, and assesses children’s perceptions of parental warmth and rejection across four dimensions: Warmth/Affection, Hostility/Aggression, Indifference/Neglect, and Undifferentiated Rejection. Items are rated on a 4-point Likert scale ranging from 1 (Almost Never True) to 4 (Almost Always True). Higher total scores indicate greater perceived parental rejection. In the present study, internal consistency was high for both parental forms (Cronbach’s α = 0.88 for mother report and 0.89 for father report).

Teacher Acceptance–Rejection Questionnaire (TARQ; Short Form). Students’ perceptions of teacher relationship quality were measured using the TARQ Short Form (Rohner, 2004; Rohner et al., 2005). The instrument contains 24 acceptance–rejection items and 4 behavioral-control items rated from 1 (Almost Never True) to 4 (Almost Always True), with higher scores indicating greater perceived rejection or control. The Greek wording was reviewed by two bilingual educational psychologists, back-translated by an independent translator, and cognitively pre-tested with 20 students aged 10–14 years. A correlated five-factor CFA showed acceptable fit, χ^2^(340) = 512.6, *p* < 0.001, CFI = 0.942, TLI = 0.936, RMSEA = 0.033, 90% CI [0.027, 0.039], and SRMR = 0.052; standardized loadings ranged from 0.48 to 0.79. Internal consistency was good for the total acceptance–rejection score (Cronbach’s α = 0.88).

Children’s Depression Inventory (CDI): Depressive symptoms were assessed using the Children’s Depression Inventory (CDI) (Kovacs, 2015), a widely used self-report screening measure for children and adolescents aged 7–17. The CDI has previously been used to screen depressive symptoms in a nationwide Greek school-based sample (Giannakopoulos et al., 2009). The CDI consists of 27 items capturing affective, cognitive, and interpersonal aspects of depressive symptomatology, including low mood, anhedonia, negative self-evaluations, and social withdrawal. Each item presents three statements of increasing severity, from which participants select the statement that best reflects their experiences over the past two weeks. Total scores range from 0 to 54, with higher scores indicating more severe depressive symptoms. Consistent with prior research, a cut-off score of 19 was used to indicate elevated symptomatology. In the present study, internal consistency was acceptable (Cronbach’s α = 0.77). In line with SEL-oriented frameworks, CDI scores were treated as screening indicators of depressive-symptom severity rather than as diagnostic classifications. Their associations with emotional self-management were examined within an SEL-oriented framework, without treating depressive symptoms and low social–emotional competence as interchangeable constructs.

Teacher’s Assessment Questionnaire of Student Functioning (TEACH). The TEACH was developed for this study through a structured content-mapping procedure based on CBCL–TRF domains (Achenbach, 2017). Two educational psychologists and one special-education specialist independently reviewed candidate items for developmental appropriateness, classroom observability, construct coverage, and redundancy. Disagreements were resolved by consensus. Items were retained only when they mapped clearly onto internalizing or externalizing school functioning and could be rated without access to the students’ CDI or RSCA responses, thereby reducing direct measurement overlap with the study outcome and proposed mediators. Teachers rated each difficulty item from 0 (Not true), through 1 (Sometimes true), to 2 (Often true).

(a) Four items capturing information relevant to SEN identification (attendance in inclusion classes, presence of an official diagnosis, somatic health conditions, and school performance), and
(b) Twenty-six items assessing internalizing (Anxious/Depressed, Withdrawn/Depressed) and externalizing (Attention Problems, Rule-Breaking Behavior, Aggressive Behavior) domains.

Total internalizing and externalizing scores were computed by summing the relevant items, with higher scores indicating greater functional difficulty. For students without formal documentation, the classification rule required an internalizing score ≥ 14 or an externalizing score ≥ 18, corresponding to the upper quartile of the study-sample distribution. The TEACH showed excellent total-scale reliability (α = 0.92; subscale αs > 0.80). A one-factor CFA of the internalizing/externalizing item pool showed adequate fit, CFI = 0.97, TLI = 0.96, RMSEA = 0.05, SRMR = 0.044, with standardized loadings from 0.54 to 0.83. The TEACH threshold rule was cross-tabulated against KEDASY documentation in the full sample (N = 465; Table S4), using documentation as a pragmatic comparator rather than an independent clinical gold standard. Of the 94 documented students, 75 met the TEACH threshold and 19 did not; of the 371 without documentation, 66 met the threshold and 305 did not. Thus, sensitivity was 75/94 = 0.80, specificity was 305/371 = 0.82, positive predictive value was 75/141 = 0.53, and negative predictive value was 305/324 = 0.94. These values evaluate the TEACH screening rule, not the final combined SEN classification, which included all 94 documented students regardless of TEACH score and the 66 additional students who met the threshold. The formally documented-only sensitivity analysis described below was used to evaluate possible criterion contamination arising from the TEACH-based classification route.

Emotional Reactivity—Resiliency Scales for Children and Adolescents (RSCA). Emotional reactivity was assessed using the 20-item RSCA Emotional Reactivity scale (Thorne & Kohut, 2007), rated from 0 (Never) to 4 (Almost Always). The scale yields Sensitivity, Recovery, and Impairment subscale scores and a total score; higher scores indicate greater emotional reactivity or regulatory difficulty. The Greek version followed forward translation, bilingual expert review, independent back-translation, cognitive pre-testing with 20 students, and final reconciliation. The theorized three-factor CFA showed acceptable fit, χ^2^(167) = 215.4, *p* = 0.007, CFI = 0.956, TLI = 0.948, RMSEA = 0.025, 90% CI [0.015, 0.033], and SRMR = 0.046; loadings ranged from 0.51 to 0.82. Internal consistency was excellent for the total score (α = 0.92) and good for Sensitivity (ω = 0.88), Recovery (ω = 0.86), and Impairment (ω = 0.91). The total REA score was used descriptively and was not entered simultaneously with its component subscales in the path models.

### 2.3. Procedures

The study was conducted in public primary and lower secondary schools in Heraklion, Chania, and Rethymnon. School counselors and principals within this defined sampling region received written study information, after which participating schools nominated liaison staff and scheduled data-collection sessions. Researchers used standardized administration scripts. Lower-secondary students completed the measures in one class period and primary students in two shorter periods. Teachers completed TEACH forms independently and did not have access to students’ self-reports. Ethical approval was obtained from the Institute of Educational Policy of the Greek Ministry of Education and the Ethics Committee of the University of Crete. Written parental or guardian consent and verbal child assent were obtained. Students were told that participation was voluntary and that they could stop without consequence. Students scoring at or above the CDI cutoff (≥19)—19.3% of the SEN group and 10.5% of the non-SEN group—were referred to the school counseling service under a written safeguarding protocol; the responsible counselor coordinated parent notification and follow-up while preserving confidentiality.

### 2.4. Statistical Analyses

**Figure 1:**
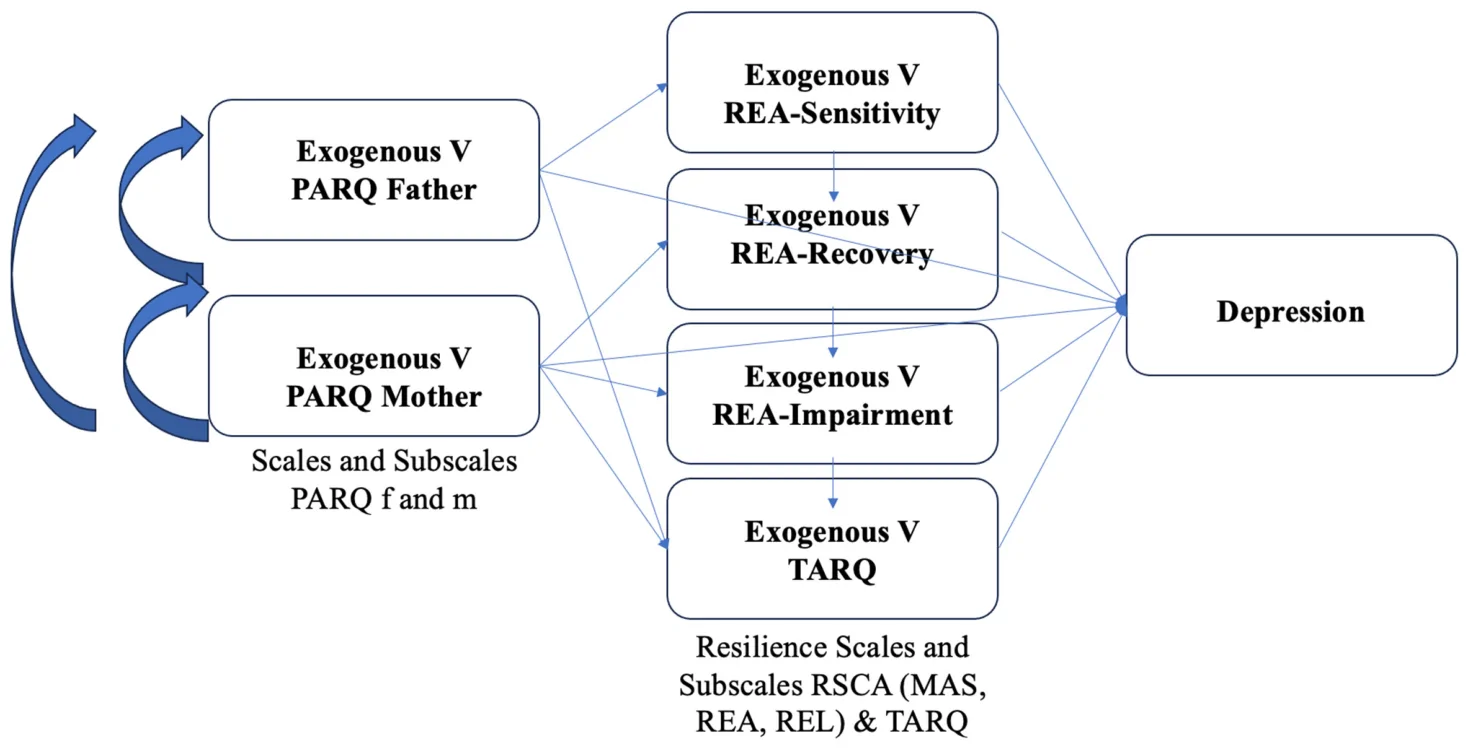
Conceptual path model depicting associations between perceived parental (mother and father) and teacher acceptance–rejection, emotional reactivity components, and depressive symptoms. Emotional reactivity is represented by sensitivity, recovery, and emotion-related functional impairment, and depressive symptoms are conceptualized as indicators of social–emotional competence outcomes.

Preliminary analyses were performed in IBM SPSS Statistics 22.0. Reliability was evaluated using Cronbach’s α and, where specified, McDonald’s ω. Missingness was reported by measure: TEACH = 0.0%, CDI = 0.4%, TARQ = 0.9%, PARQ-mother = 1.1%, RSCA = 1.5%, and PARQ-father = 3.6%. Little’s MCAR test was non-significant, χ^2^(50) = 54.0, *p* = 0.32. Descriptive bivariate correlations in Table 1 and Table 2 were calculated in SPSS using available observations for each variable pair (pairwise deletion), so their contributing cases can differ by pair (see also Figure 1). In contrast, the path models used full information maximum likelihood (FIML) on the model variables; incomplete mother/father reports did not require listwise deletion. The pairwise descriptive matrices are not the Mplus FIML analysis covariance matrices used to estimate or evaluate the reduced models. Absolute skewness was ≤1.18 and absolute kurtosis ≤ 1.42. Four cases exceeded the conservative Mahalanobis-distance criterion (*p* < 0.001), but no Cook’s distance exceeded 1.0 and excluding these cases did not materially alter the results. Preliminary group comparisons were treated as exploratory and controlled using the Benjamini–Hochberg false-discovery-rate procedure at q = 0.05; both raw *p* values and adjusted q values are reported.

Path models were estimated in Mplus 8.10 using robust maximum likelihood (MLR), FIML, and TYPE = COMPLEX with teacher as the clustering variable. A school-cluster sensitivity analysis yielded substantively equivalent coefficients. The conceptual model considered paths from maternal, paternal, and teacher rejection to emotional reactivity and depressive symptoms, paths from emotional-reactivity components to depressive symptoms, and covariances among rejection measures and emotional-reactivity residuals. The reported group-specific fits used the reduced specifications itemized in Table S5: six freely estimated ON paths without SEN and eight in the combined SEN group, four free variances (Sensitivity, Recovery, Impairment, and depressive symptoms), and four free WITH covariances (maternal with paternal rejection and the three pairs of emotional-reactivity components) in each group. Maternal and paternal rejection variances and the teacher-rejection residual variance were fixed in the inputs. Other candidate ON paths and residual covariances were fixed at zero in these reduced specifications. The REA total score was not entered with its subscales. Sex, age, and grade level were included in adjusted sensitivity models. Indirect effects were tested using 5000 bias-corrected bootstrap resamples and are reported with unstandardized b, bootstrap SE, standardized β, exact p, and 95% confidence intervals. R^2^ values are identified by their source specification in the Section 3.

**Figure 2:**
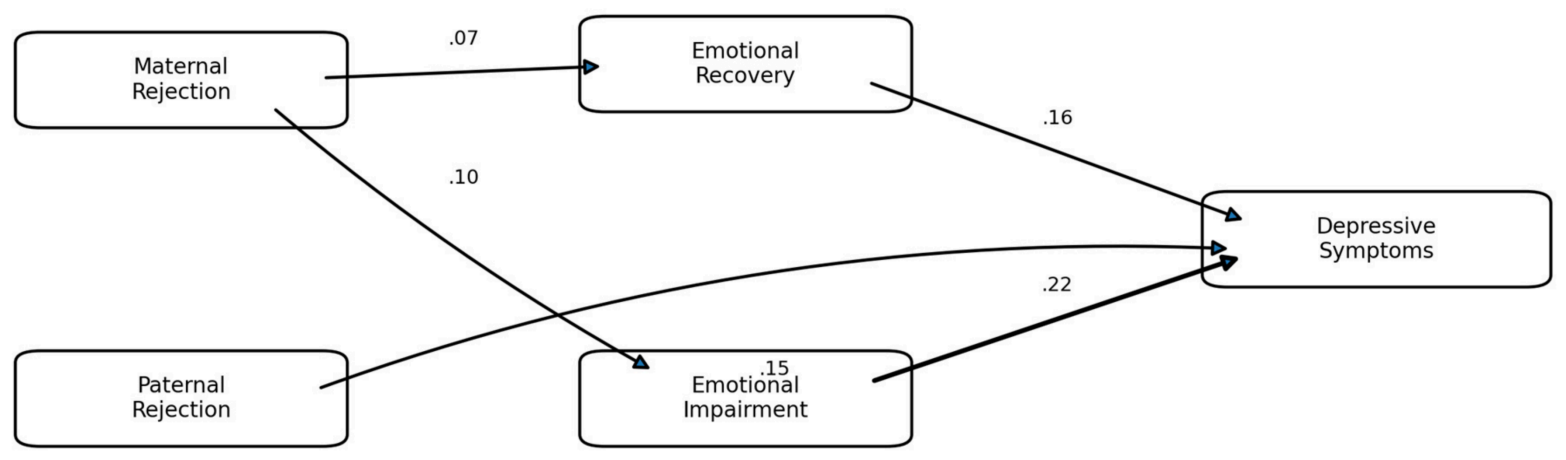
Standardized structural paths for students without SEN. For legibility, the diagram shows significant freely estimated paths only. Other modeled variables and paths are not displayed; an absent arrow may represent either a nonsignificant free path or a path constrained to zero in this reduced model (see Section 2.4). Bivariate correlations among rejection measures and REA subscales are reported in Table 1, and indirect effects are reported in Table S2. The three estimated covariances among emotional-reactivity components are omitted from this diagram and listed in Table S5. Depressive-symptom R^2^ in the Section 3 is from a separate expanded outcome specification, not the selected-arrow diagram.

**Figure 3:**
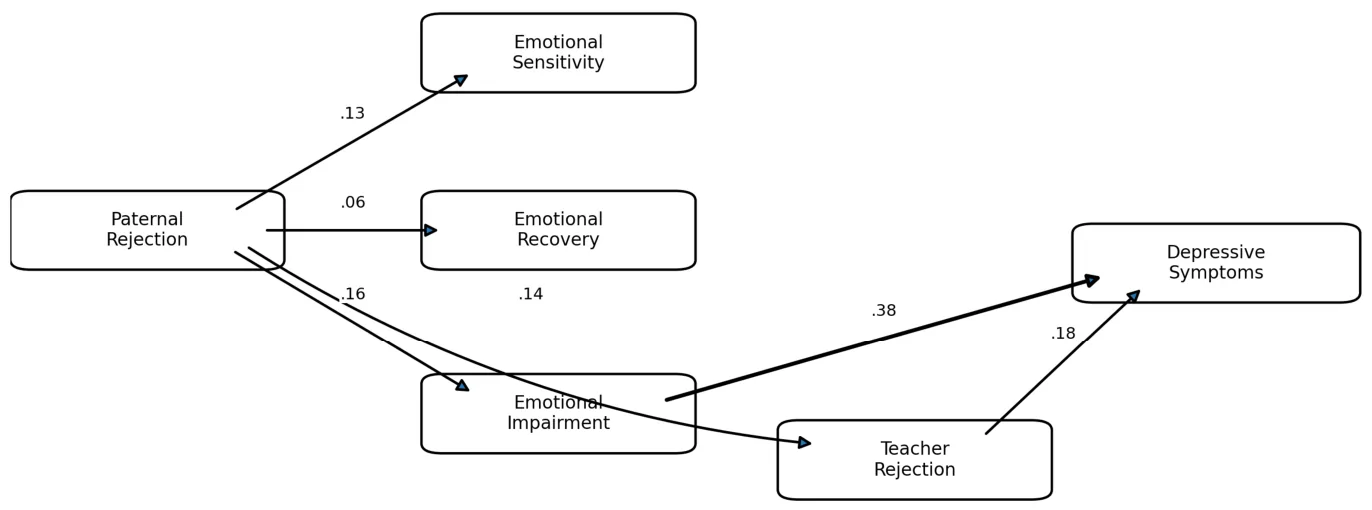
Selected structural paths for students with SEN. For legibility, the diagram shows selected paths. The paternal rejection–Sensitivity arrow (b = 0.13) comes from the separate indirect-effect specification, whereas other shown paths summarize the reduced fit. Other modeled variables and paths are not displayed; an absent arrow may represent either a nonsignificant free path or a path constrained to zero (see Section 2.4). Bivariate correlations among rejection measures and REA subscales are reported in Table 2, and indirect effects are reported in Table S2. Coefficient notation: the 0.13 arrow for paternal rejection–Sensitivity and the 0.14 arrow for paternal rejection–teacher rejection denote unstandardized b, not standardized β; see Section 3.3. The three estimated covariances among emotional-reactivity components are omitted from this diagram and listed in Table S5. Depressive-symptom R^2^ in the Section 3 is from a separate expanded outcome specification, not the selected-arrow diagram.

Configural multigroup models were followed by one-degree-of-freedom equality constraints for each principal structural path. A path was interpreted as differing between groups when the constrained model worsened by a significant Δχ^2^ test and ΔCFI was at least 0.010 in magnitude. The covariance matrix of the seven observed variables supplied 28 distinct moments (seven variances and 21 covariances). As detailed in Table S5, the reduced models contained 14 and 16 free parameters in students without SEN and the combined SEN group, respectively (six or eight ON paths plus four free variances and four free WITH covariances), yielding the reported 14 and 12 model degrees of freedom. The equality tests in Table S3 used a separate expanded configural specification with each compared path estimable in both groups; the reduced group-specific fit statistics do not describe that expanded comparison model. Figure 2 and Figure 3 summarize selected paths and omit some freely estimated paths; Figure 3 also displays the paternal rejection—Sensitivity coefficient from the separate indirect-effect specification identified in its legend. No independent cross-validation was undertaken. The primary structural paths and indirect effects were treated as theory-driven primary analyses; ancillary correlations and unplanned subgroup comparisons were identified as exploratory. A sensitivity analysis restricted the SEN group to the 94 students with KEDASY-documented status to evaluate criterion contamination. Supplementary Table S7 reports coefficients for every free structural ON path in the reduced models, including nonsignificant estimates. Paths fixed to zero are identified in Table S5. Paths fixed to zero are listed in Table S5. 

## 3. Results

### 3.1. Preliminary Group Comparisons

Preliminary comparisons are summarized in Supplementary Table S1. Students with SEN reported higher depressive symptoms than students without SEN, t(463) = 8.39, *p* < 0.001, q < 0.001, d = 0.82. Differences in parental and teacher relationship dimensions were generally small to moderate, whereas differences in REA recovery and impairment were moderate and small, respectively. Emotional sensitivity did not differ between groups, *p* = 0.590, q = 0.590. All statistically significant comparisons reported below remained significant after false-discovery-rate adjustment.

Students with SEN reported lower paternal and maternal warmth and higher hostility, neglect, and undifferentiated rejection than students without SEN. They also reported higher teacher hostility, neglect, undifferentiated rejection, and behavioral control. Raw and FDR-adjusted significance levels, confidence intervals, and effect sizes for both groups are provided in Table S1, avoiding reliance on p values alone.

**Figure 4:**
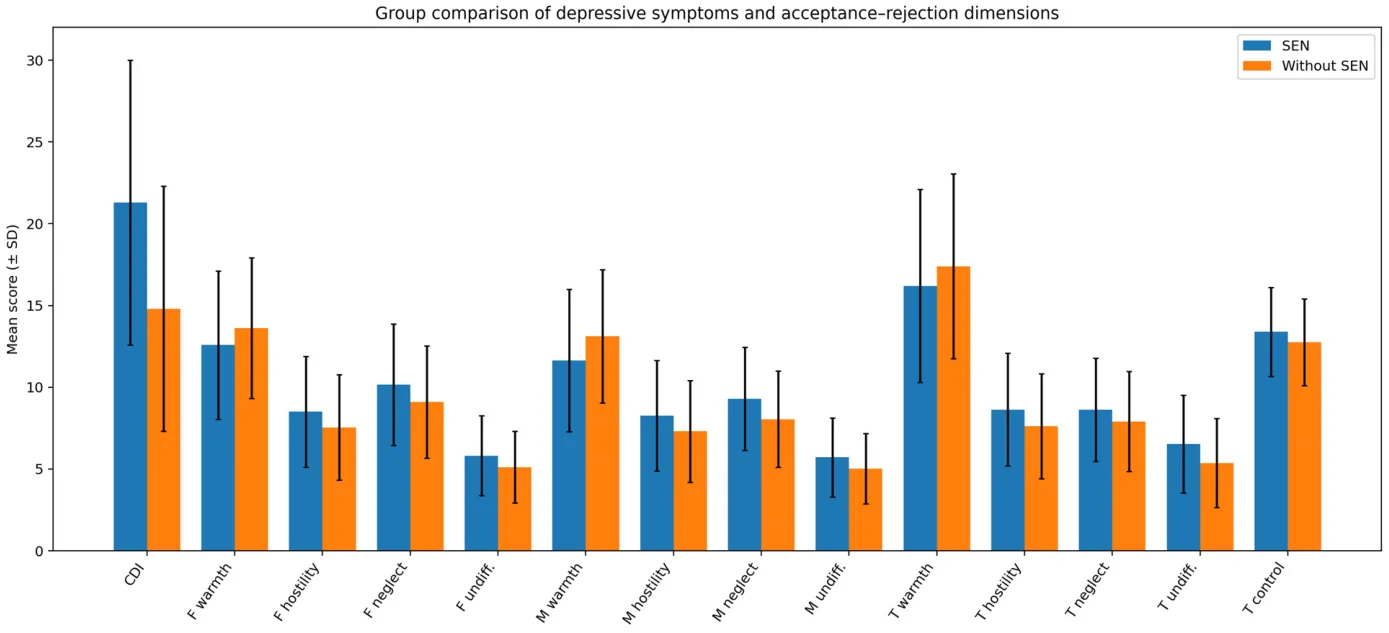
Mean scores (±SD) for depressive symptoms and perceived parental and teacher acceptance–rejection dimensions in students with SEN and without SEN. The figure displays both groups directly; complete test statistics, confidence intervals, effect sizes, and FDR-adjusted q values are reported in Supplementary Table S1.

Students with SEN showed greater emotion-related impairment (M = 16.67, SD = 9.39) than students without SEN (M = 13.94, SD = 8.28), t(463) = 3.22, *p* = 0.001, q = 0.004, d = 0.32, and longer recovery times (M = 4.61, SD = 3.84 versus M = 2.90, SD = 3.51), t(463) = 4.83, *p* < 0.001, q < 0.001, d = 0.47. Sensitivity did not differ significantly. Figure 4 displays both groups directly; Table S1 provides the complete numerical comparison.

Pairwise available-case bivariate associations are presented separately for students without SEN (Table 1) and students with SEN (Table 2), making group placement unambiguous. Their correlation matrices differ in case handling from the FIML path analyses and should not be used to infer the reduced-model standardized coefficients or fit indices. In students without SEN, Recovery correlated more strongly with maternal rejection (r = 0.226, *p* < 0.01) than with paternal rejection (r = 0.167, *p* < 0.01). In students with SEN, the total REA score correlated with paternal rejection (r = 0.332, *p* < 0.01) and maternal rejection (r = 0.285, *p* < 0.01). The total REA score and its subscales are shown together only for descriptive transparency; the total score was excluded from path models containing Sensitivity, Recovery, and Impairment.

### 3.2. Path Analysis: Students Without SEN

The first path model examined theory-driven associations among parental and teacher rejection, REA components, and depressive symptoms in students without SEN. Fit of the reduced model described above was excellent, χ^2^(14) = 18.36, *p* = 0.19, CFI = 0.989, TLI = 0.978, RMSEA = 0.026, 90% CI [0.000, 0.055], SRMR = 0.034, and AGFI = 0.95. The reduced model explained 7% of the variance in perceived teacher rejection. The depressive-symptom R^2^ of 0.28 came from a separate expanded equation with paternal rejection (β = 0.150), Recovery (β = 0.160), Impairment (β = 0.220), age (β = 0.221), and biological sex (β = 0.284445; male coded 1) as predictors (Table S8); it is not an R^2^ value from the reduced six-path fit or Figure 3. The Recovery R^2^ of 0.06 refers to a separate expanded equation with maternal, paternal, and teacher rejection as predictors. The Impairment R^2^ of 0.09 refers to a further expanded equation that also included age (β = 0.221). In a three-rejection-predictor calculation from the rounded correlations in Table 1, Impairment R^2^ was 0.041; age contributed approximately 0.049 additional explained variance in the expanded analysis, bringing R^2^ to 0.090 (Table S6). Neither expanded equation is the reduced six-path model shown in Figure 3. Maternal rejection was associated with teacher rejection in the reduced model (standardized β = 0.26), consistent with the teacher-rejection R^2^ of 0.07; this estimated path is omitted from the summary diagram. The covariate-adjusted model produced the same substantive pattern.

For a descriptive check against the published bivariate data, Supplementary Table S6 gives simultaneous standardized three-predictor coefficients calculated from Table 1 and Table 2. These correlation-derived coefficients and implied R^2^ values use the pairwise available-case descriptive matrices, not the Mplus FIML analysis matrices; they are not coefficients or fit statistics from the reduced structural models in Figure 3 and Figure 4. For example, maternal rejection and Recovery correlate at r = 0.226 in Table 1 whereas their reduced-model path is β = 0.07 (Table S7); paternal rejection and Recovery correlate at r = 0.159 in Table 2 whereas their reduced-model path is β = 0.06. Those estimates have different analytic provenance and should not be substituted for one another.

Within the non-SEN model, maternal rejection was associated with Recovery and Impairment, and Impairment was associated with depressive symptoms (β = 0.22, *p* < 0.001). Paternal rejection showed a direct association with depressive symptoms (β = 0.15, *p* = 0.031). The specific maternal-rejection indirect effects through Recovery and Impairment were both statistically different from zero (Table S2). No teacher-rejection indirect effect was interpreted as statistically supported. Figure 2 is a summary of significant paths, not a complete representation of the fitted specification. Statistically significant standardized path coefficients are shown in Figure 2.

### 3.3. Path Analysis: Students with SEN

The second path model tested the same theory-driven framework in students with SEN. Fit of the reduced model described above was excellent, χ^2^(12) = 6.81, *p* = 0.87, CFI = 1.00, TLI = 1.00, RMSEA = 0.000, 90% CI [0.000, 0.042], SRMR = 0.024, and AGFI = 0.96. Correlations among the REA subscales were consistent with Table 2 (Sensitivity–Recovery r = 0.475, Sensitivity–Impairment r = 0.699, Recovery–Impairment r = 0.573). VIFs ranged from 1.51 to 2.29 and tolerances from 0.44 to 0.66, indicating that multicollinearity was not problematic when the REA total score was excluded. In the reduced model, paternal rejection–Sensitivity was small and statistically uncertain (b = 0.0245, SE = 0.0400, β = 0.049, *p* = 0.540; Table S7). In the separate indirect-effect specification used for Table S2, the coefficient was b = 0.13 (*p* = 0.008). In the reduced fit, paternal rejection–Recovery was β = 0.06 (*p* = 0.041), and teacher rejection (β = 0.18, *p* = 0.023) and Impairment (β = 0.38, *p* < 0.001) were associated with depressive symptoms. The reduced model explained 11% of the variance in teacher rejection. The depressive-symptom R^2^ of 0.31 came from a separate expanded equation with Sensitivity (β = 0.064794), Recovery (β = 0.202481), Impairment (β = 0.307771), teacher rejection (β = 0.145786), age (β = 0.100), and biological sex (β = 0.100; male coded 1) as predictors (Table S8); it is not an R^2^ value from the reduced eight-path fit or Figure 3. Separate expanded equations that additionally included maternal, paternal, and teacher rejection and age explained 8% of Sensitivity, 6% of Recovery, and 15% of Impairment. Age had standardized coefficients of β = 0.136, 0.096, and 0.261, respectively, with corresponding incremental R^2^ values of 0.01854, 0.00915, and 0.06823 over the three-rejection-predictor calculations (Table S6). These three emotional-reactivity R^2^ values refer to the expanded analyses rather than the reduced eight-path model in Figure 3. In the reduced teacher-rejection equation, paternal rejection was the sole predictor: its arrow label in Figure 3 is the unstandardized coefficient b = 0.14, whereas its STDYX-standardized coefficient was approximately β = 0.33. The latter is consistent with the reported teacher-rejection R^2^ of 0.11. Two additional freely estimated paths from Sensitivity (b = 0.092, SE = 0.067, β = 0.08, *p* = 0.170) and Recovery (b = 0.570, SE = 0.315, β = 0.25, *p* = 0.070) to depressive symptoms were not individually statistically significant; they are therefore omitted from the selected-path diagram in Figure 3. Specific indirect effects are reported in Table S2 and should not be conflated with tests of individual component paths. The paternal rejection–teacher rejection–depressive symptoms indirect effect in Table S2 was estimated in a separate indirect-effect specification: teacher rejection–depressive symptoms was b = 0.186 (β = 0.13) there, compared to β = 0.18 in the reduced model shown in Figure 4. In the analysis restricted to the 94 students with KEDASY-documented status, teacher rejection (β = 0.17, *p* = 0.041) and Impairment (β = 0.34, *p* < 0.001) remained associated with depressive symptoms. The paternal rejection–Recovery estimate was similar to the combined-group estimate (β = 0.05 versus 0.06), but less statistically certain in the smaller documented-only sample (*p* = 0.081). The combined-group paternal finding should therefore not be assumed to hold for documented SEN alone.

Formal multigroup tests used an expanded configural model, distinct from the reduced group-specific fits in Section 3.2 and Section 3.3, with each compared path freely estimated in both groups; one path at a time was then constrained equal across groups. Evidence of a group difference required both a significant Δχ^2^ test and an absolute ΔCFI of at least 0.010. As shown in Table S3, the maternal rejection—Recovery, maternal rejection—Impairment, and teacher rejection—depressive symptoms paths differed across groups. The paternal rejection—Recovery constraint produced a significant Δχ^2^ but did not reach the prespecified ΔCFI criterion and was therefore interpreted as inconclusive. Other apparent group differences were treated descriptively.

Taken together, the results support group differences for a limited subset of theory-driven paths rather than a global claim that every coefficient differed. Maternal rejection showed stronger associations with selected emotional-reactivity dimensions in students without SEN, whereas the teacher-rejection association with depressive symptoms was stronger in students with SEN. Other contrasts, including several paternal-rejection paths, remain descriptive or inconclusive and should not be interpreted as statistically established group differences. These comparisons use the combined SEN group, including 66 students identified solely by teacher screening.

## 4. Discussion

The present study examined cross-sectional associations among perceived parental and teacher rejection, emotional reactivity, and depressive symptoms in students with and without SEN. The findings identify potential explanatory pathways but do not establish temporal order or causal mechanisms. Formal multigroup tests supported differences for maternal rejection—Recovery, maternal rejection—Impairment, and teacher rejection—depressive symptoms, whereas other apparent contrasts were descriptive or inconclusive. Reciprocal explanations remain plausible: depressive symptoms may heighten sensitivity to rejection, perceived rejection may be associated with regulatory difficulty, and both may reflect unmeasured family, school, or individual factors.

### 4.1. Group Differences and Learner Variability

Consistent with prior research, students with SEN reported higher depressive symptoms, greater perceived rejection, poorer recovery, and greater emotion-related impairment. These findings describe average differences in a combined, heterogeneous group, 41.3% of whom were identified solely by teacher screening, and should not be generalized to all students with documented SEN. Emotional reactivity may be one correlate linking relational adversity and psychological distress, but the cross-sectional design and shared self-report method prevent it from being identified as a causal mechanism.

### 4.2. Pathways Among Students Without SEN

Among students without SEN, paternal rejection had a direct statistical association with depressive symptoms, whereas maternal rejection had indirect associations through Recovery and Impairment. The maternal pathway should therefore be interpreted as central to the reactivity-related indirect association, not as evidence that maternal relationships were uniformly more important than paternal relationships. Because all variables were measured concurrently, the indirect effects represent model-consistent associations rather than temporal mediation.

These pathways are consistent with a large body of research demonstrating that unsupportive or hostile parenting is associated with poorer emotion regulation and increased risk for depressive symptomatology in children and adolescents (Bodner et al., 2018; Morris et al., 2007, 2017; Yuan & Wang, 2016). Maternal emotion socialization practices—such as invalidating responses, minimization of emotional expression, or limited emotional awareness—have been repeatedly linked to emotion dysregulation and internalizing difficulties (Buckholdt et al., 2014; Katz et al., 2014; Shortt et al., 2016). This is consistent with evidence that parents’ own sensory processing sensitivity and reactivity shape how children attend to and process emotionally salient information, indicating that emotional reactivity is transmitted, at least in part, through the relational and attentional dynamics of the parent–child dyad (Christou et al., 2024; Christou et al., 2025). From an attachment perspective, securely attached children are more likely to internalize effective emotion regulation strategies and deploy them across relational contexts, whereas insecure attachment is associated with regulatory difficulties and heightened psychological distress (Brumariu & Kerns, 2010; Mallers et al., 2010).

Notably, in students without SEN, perceived maternal rejection was also strongly associated with perceived teacher rejection, suggesting continuity in relational representations across home and school contexts. This pattern supports models proposing that children generalize expectations and interpretations of relationships with primary caregivers to interactions with other significant adults, including teachers (Allen et al., 2003; Riley, 2010).

### 4.3. Pathways Among Students with SEN

In the combined SEN group, paternal rejection was associated with Recovery in the reduced fit; its Sensitivity estimate in that fit was small and statistically uncertain (β = 0.049, *p* = 0.540). An association with Sensitivity was observed in the separate indirect-effect specification. Teacher rejection and Impairment were associated with depressive symptoms in the reduced fit. The formal multigroup analysis supported a stronger teacher-rejection association with depressive symptoms in this group, but did not confirm every apparent paternal-path difference. Accordingly, the SEN findings are best described as a pattern of school- and family-relational correlates rather than as pathways originating exclusively from paternal rejection.

Contrary to our tentative expectation, maternal rejection did not show a direct or indirect association with depressive symptoms in the SEN model. This null pattern should not be interpreted as evidence that maternal relationships are unimportant; it may reflect shared variance with paternal and teacher rejection, heterogeneity within the broad SEN group, or limited power to detect smaller component-specific associations. Replication in larger and more diagnostically homogeneous samples is needed.

Although research on paternal influences in students with SEN remains limited, existing evidence suggests that fathers’ emotional availability, involvement, and emotion socialization practices play a distinct and sometimes stronger role than maternal behaviors in shaping children’s emotional competence (Denham & Kochanoff, 2002; Gregory et al., 2020). Fathers’ responses to children’s emotions have been linked to emotion regulation, problem-solving abilities, and adaptation to stress (Easterbrooks & Goldberg, 1990; Roberts & Strayer, 1987). For children with SEN, paternal stress related to caregiving demands, emotional adjustment, and economic burden may further compromise fathers’ capacity to provide consistent emotional support (Ahmad & Dardas, 2015; Di Giulio et al., 2014; Paynter et al., 2018). This convergence is consistent with recent evidence that perceived paternal rejection in preadolescents with SEN is associated with diminished self-perception and greater vulnerability to peer victimization, further underscoring the centrality of the father–child relationship for this population (Christou et al., 2026c).

Cultural factors may also contribute to these patterns. In the Cretan context, traditional norms surrounding masculinity and emotional expression may discourage fathers from modeling adaptive emotion regulation or openly expressing vulnerability, potentially reinforcing suppressive or hostile emotional styles (Makri-Botsari & Karagianni, 2014). Such patterns have been associated with increased rumination, difficulty decoding emotions, and elevated depressive symptoms in youth (Rieffe & De Rooij, 2012). When combined with the heightened emotional needs of children with SEN, these dynamics may amplify emotional dysregulation and relational strain. Because cultural variables such as masculinity norms and paternal emotional-expression styles were not directly measured in the present study, this explanation should be regarded as a hypothesis for future research rather than a finding, and is offered here only as one plausible interpretive frame among others.

The pathway linking paternal and teacher rejection further aligns with IPARTheory, which posits that children’s perceptions of acceptance or rejection by significant others exert a powerful influence on psychological adjustment (Khaleque, 2017). Students with SEN may be particularly sensitive to relational cues and more dependent on consistent affirmation from adults, increasing the likelihood that negative relational experiences generalize across contexts (Henricsson & Rydell, 2004; Pianta & Stuhlman, 2004; Prino et al., 2016).

One notable finding was the direct association between perceived teacher rejection and depressive symptoms in the combined SEN group, which the formal multigroup analysis indicated was stronger than in students without SEN. The teacher-rejection association was also present in the documented-only analysis; the reported between-group test, however, used the combined SEN group. This extends attachment-informed perspectives that have traditionally foregrounded parent–child relationships by identifying teachers as an additional, developmentally salient relational context. The finding does not demonstrate that teachers are more influential than parents or that school relationships replace family relationships; rather, it indicates that teacher–student relationships may make a distinct contribution to the emotional adjustment of students with SEN. This interpretation should be tested in longitudinal and intervention studies and across more homogeneous diagnostic or dimensional subgroups.

### 4.4. Implications for SEL and Inclusive Practice

Taken together, the findings suggest that inclusive SEL research should attend to learner variability and to both family and school relationships. However, the data do not establish that universal SEL approaches are ineffective or that specific differentiated interventions will improve outcomes. The present results support hypotheses for future intervention research rather than immediate program prescriptions.

The differentiated pattern across emotional-reactivity components is also important. Impairment showed the most consistent direct association with depressive symptoms, whereas Recovery and Sensitivity were involved in more group-specific relational pathways. Emotional reactivity should therefore not be treated as a unitary construct when developing or evaluating relational and social–emotional supports.

As an observational, cross-sectional study, the present findings cannot establish whether targeted professional development for teachers focusing on emotional responsiveness and relationship-building, or family-focused interventions engaging fathers around paternal stress and emotion socialization practices, would in fact improve outcomes for students with SEN. These are offered as tentative, theoretically motivated implications for practice rather than evidence-based recommendations, and their effectiveness would need to be established through dedicated intervention research (e.g., randomized or quasi-experimental designs) before being adopted.

Translating these directions into practice also requires attention to barriers that may limit fathers’ participation, including work schedules, caregiving demands, and limited access to school-based services. Future intervention studies could therefore evaluate flexible or hybrid parent-support programs, school-mediated outreach to fathers, and family–school partnerships that combine teacher relationship-building with support for parental stress and emotion socialization. These delivery approaches are hypotheses prompted by the observed associations and require empirical evaluation; the similar-sized paternal rejection–Recovery estimate was less statistically certain in the smaller documented-only analysis.

### 4.5. Limitations and Directions for Future Research

Several limitations should be considered when interpreting the findings of the present study. First, the cross-sectional design precludes causal inferences regarding the directionality of associations among perceived acceptance–rejection, emotional reactivity, and depressive symptoms. Although the proposed pathways are theoretically grounded, longitudinal designs are needed to clarify developmental sequencing and reciprocal influences over time.

Second, the study relied primarily on self-report measures for children’s perceptions of parental and teacher acceptance–rejection, emotional reactivity, and depressive symptoms. While children’s subjective perceptions are central to social–emotional functioning, shared method variance and reporting biases cannot be ruled out. This is a common-method-variance concern in a particularly direct form: the predictors (perceived parental and teacher rejection), the proposed mediators (emotional reactivity), and the outcome (depressive symptoms) were all drawn from the same student self-report, collected in the same session. Teacher report (TEACH) was used only to inform SEN group classification and did not provide an independent source for the predictor, mediator, or outcome variables themselves, and therefore does not resolve this concern. Shared reporter and shared method can inflate the observed associations among these variables, and the true magnitude of the reported pathways should be interpreted with this in mind. Future research would benefit from multi-method and multi-informant approaches, including observational data, parent and teacher reports of emotion socialization practices, and physiological indicators of emotional reactivity. Recent work demonstrating that parent–child covariation in behavioral and physiological reactivity to threat and distress predicts children’s aggression suggests that dyadic, multi-method designs of this kind can uncover regulatory mechanisms not captured by self-report alone, and may similarly illuminate the pathways to depressive symptoms identified here (Fanti et al., 2026; Christou et al., 2026a, 2026b).

Third, the SEN classification combined 94 KEDASY-documented students with 66 students identified through TEACH functional screening (41.3% of the combined group). The latter cannot be described as having documented SEN. This reflects inclusive school practice but creates heterogeneity and possible criterion contamination because TEACH internalizing difficulties overlap conceptually with depressive symptoms. In the formally documented-only sensitivity analysis (n = 94), teacher rejection remained associated with depressive symptoms (β = 0.17, *p* = 0.041) and Impairment remained significant (β = 0.34, *p* < 0.001), while the paternal rejection–Recovery estimate was similar to that in the combined SEN group (β = 0.05 versus 0.06) but less statistically certain in the smaller documented-only sample (*p* = 0.081). This pattern reduces but does not eliminate concern about classification circularity, and the subgroup analysis should be interpreted cautiously.

Several additional methodological constraints should be considered. Students were nested within teachers and schools; teacher-level cluster-robust estimation and a school-cluster sensitivity analysis were therefore used, but 26 schools provide limited precision for higher-level variance estimates. A separate covariate-adjusted sensitivity specification for sex, age, and grade produced the following SEN-group estimates (teacher rejection β = 0.16, *p* = 0.032; Impairment β = 0.35, *p* < 0.001; paternal rejection—Sensitivity β = 0.12, *p* = 0.008; depression R^2^ = 0.29); its paternal rejection—Sensitivity coefficient should not be read as the corresponding reduced-model coefficient (β = 0.049, *p* = 0.540). VIFs of 1.51–2.29 indicated acceptable multicollinearity only after excluding the REA total score from the structural model. Exploratory group comparisons were FDR-adjusted, but the number of model coefficients still increases the possibility of chance findings. Finally, only three principal paths met both multigroup criteria; other between-group contrasts should remain descriptive.

Fourth, cultural context may limit the generalizability of the findings. The study was conducted in schools in Greece, where family roles, gender norms, and emotional socialization practices may differ from those in other cultural settings. Cross-cultural replication studies are needed to determine the extent to which the observed maternal, paternal, and teacher pathways generalize across sociocultural contexts.

Future research should also explore developmental timing and transitions, such as the shift from childhood to adolescence, during which emotional reactivity and relational influences may change in salience. Longitudinal and intervention-based studies examining how school-based social–emotional learning programs can be adapted to address learner variability—particularly for students with heightened emotional reactivity or SEN—are especially warranted. Finally, greater attention to the role of fathers and other caregivers in emotion socialization, as well as the interaction between family and school contexts, may yield important insights for designing more inclusive and responsive SEL frameworks.

## 5. Conclusions

Comparative examination of the models indicated three statistically supported group differences—maternal rejection—Recovery, maternal rejection—Impairment, and teacher rejection—depressive symptoms—and several descriptive or inconclusive contrasts. These tests compared students without SEN with the combined, partly teacher-screened SEN group. In students without SEN, paternal rejection showed a direct association with depressive symptoms and maternal rejection showed indirect associations through emotional reactivity. In students with SEN, teacher rejection and Impairment showed direct associations with depressive symptoms, while paternal rejection showed several indirect associations. Emotional reactivity is therefore described as a potential explanatory pathway, not a demonstrated mechanism. The findings justify longitudinal and independently classified replication of these selected associations; they do not establish that all pathways differ between groups or that the observed indirect associations are causal mechanisms.

## Figures and Tables

**Table 1:** Bivariate correlations among perceived teacher acceptance–rejection (TARQ), perceived parental acceptance–rejection (PARQ; mother and father total scores), and emotional reactivity (REA) components for students without SEN.

Variable	TARQ	PARQ-f	PARQ-m	REA	REA Sensitivity	REA Recovery	REA Impairment
TARQ	1	0.225 **	0.280 **	0.079	0.003	0.053	0.112
PARQ-f	0.225 **	1	0.530 **	0.205 **	0.150 **	0.167 **	0.182 **
PARQ-m	0.280 **	0.530 **	1	0.191 **	0.124 *	0.226 **	0.151 **
REA	0.079	0.205 **	0.191 **	1	0.801 **	0.677 **	0.919 **
REA Sensitivity	0.003	0.150 **	0.124 *	0.801 **	1	0.379 **	0.577 **
REA Recovery	0.053	0.167 **	0.226 **	0.677 **	0.379 **	1	0.479 **
REA Impairment	0.112	0.182 **	0.151 **	0.919 **	0.577 **	0.479 **	1

Note. TARQ = Teacher Acceptance–Rejection Questionnaire; PARQ-f/PARQ-m = Parental Acceptance–Rejection Questionnaire (father/mother); REA = Emotional Reactivity. * *p* < 0.05. ** *p* < 0.01.

**Table 2:** Bivariate correlations among perceived teacher acceptance–rejection (TARQ), perceived parental acceptance–rejection (PARQ; mother and father total scores), and emotional reactivity (REA) components for students with SEN.

Variable	TARQ	PARQ-f	PARQ-m	REA	REA Sensitivity	REA Recovery	REA Impairment
TARQ	1	0.334 **	0.169 *	0.112	0.061	0.072	0.130
PARQ-f	0.334 **	1	0.470 **	0.332 **	0.049	0.159 *	0.169 *
PARQ-m	0.169 *	0.470 **	1	0.285 **	0.234 **	0.215 **	0.272 **
REA	0.112	0.332 **	0.285 **	1	0.840 **	0.729 **	0.948 **
REA Sensitivity	0.061	0.049	0.234 **	0.840 **	1	0.475 **	0.699 **
REA Recovery	0.072	0.159 *	0.215 **	0.729 **	0.475 **	1	0.573 **
REA Impairment	0.130	0.169 *	0.272 **	0.948 **	0.699 **	0.573 **	1

Note. TARQ = Teacher Acceptance–Rejection Questionnaire; PARQ-f/PARQ-m = Parental Acceptance–Rejection Questionnaire (father/mother); REA = Emotional Reactivity. * *p* < 0.05. ** *p* < 0.01.

## Data Availability

The datasets generated during and/or analyzed during the current study are available from the corresponding author on reasonable request.

## Supplementary Materials

The following supporting information can be downloaded at: https://www.mdpi.com/article/10.3390/bs16101830/s1, Tables S1–S8: Supplementary information on SEN classification, statistical model specifications, path coefficients, indirect effects, and sensitivity analyses.
