# Supplementary material for: Differential Pathways to Depressive Symptoms in Students With and Without Special Educational Needs: The Role of Emotional Reactivity and Parent–Teacher Relationships
Source: Behav Sci (Basel). 2026 Oct 7;16(10):1830. doi: 10.3390/bs16101830 (PMC13701697; doi:10.3390/bs16101830)
Supplement: Supplementary file 1 [file behavsci-16-01830-s001.zip › behavsci-4456408-supplementary.pdf]

Supplementary Tables

Supplementary Table S1. Complete descriptive and inferential comparisons between students with and without SEN.

Variable	SEN M (SD)	Without SEN M (SD)	t(463)	p / q	95% CI for difference	d
CDI depressive symptoms	21.30 (8.70)	14.80 (7.50)	8.39	<.001 / <.001	[4.98, 8.02]	0.82
Father warmth/affection	12.58 (4.53)	13.62 (4.29)	-2.44	.015 / .018	[-1.88, -0.20]	-0.24
Father hostility/aggression	8.50 (3.38)	7.55 (3.21)	2.98	.003 / .004	[0.32, 1.58]	0.29
Father indifference/neglect	10.15 (3.71)	9.10 (3.42)	3.05	.002 / .004	[0.37, 1.73]	0.30
Father undifferentiated rejection	5.82 (2.44)	5.12 (2.19)	3.15	.002 / .004	[0.26, 1.14]	0.31
Mother warmth/affection	11.63 (4.35)	13.12 (4.08)	-3.66	<.001 / .001	[-2.29, -0.69]	-0.36
Mother hostility/aggression	8.26 (3.37)	7.31 (3.11)	3.04	.003 / .004	[0.34, 1.56]	0.30
Mother indifference/neglect	9.30 (3.16)	8.05 (2.94)	4.24	<.001 / <.001	[0.67, 1.83]	0.41
Mother undifferentiated rejection	5.71 (2.42)	5.03 (2.15)	3.10	.002 / .004	[0.25, 1.11]	0.30
Teacher hostility/aggression	8.63 (3.45)	7.62 (3.20)	3.15	.002 / .004	[0.38, 1.64]	0.31
Teacher indifference/neglect	8.63 (3.16)	7.91 (3.05)	2.39	.017 / .018	[0.13, 1.31]	0.23
Teacher undifferentiated rejection	6.54 (2.99)	5.37 (2.72)	4.26	<.001 / <.001	[0.63, 1.71]	0.42
Teacher behavioural control	13.39 (2.72)	12.76 (2.65)	2.41	.016 / .018	[0.12, 1.14]	0.24
REA sensitivity	18.42 (7.20)	18.05 (6.94)	0.54	.590 / .590	[-0.98, 1.72]	0.05
REA recovery	4.61 (3.84)	2.90 (3.51)	4.83	<.001 / <.001	[1.01, 2.41]	0.47
REA impairment	16.67 (9.39)	13.94 (8.28)	3.22	.001 / .004	[1.07, 4.39]	0.32

Note. Positive differences indicate higher scores in the SEN group. q values are Benjamini–Hochberg FDR-adjusted.

Supplementary Table S2. Bootstrapped specific indirect effects (5,000 resamples).

Group	Indirect path	b	SE	95% BC CI	p	β
Without SEN	Maternal rejection - Recovery - depressive symptoms	.008	.003	[.003, .014]	.003	.011
Without SEN	Maternal rejection - Impairment - depressive symptoms	.016	.006	[.006, .030]	.006	.022
SEN	Paternal rejection - Sensitivity - depressive symptoms	.012	.007	[.002, .031]	.034	.02
SEN	Paternal rejection - Recovery - depressive symptoms	.009	.005	[.001, .022]	.041	.02
SEN	Paternal rejection - Impairment - depressive symptoms	.037	.015	[.011, .070]	.014	.06
SEN	Paternal rejection - Teacher rejection - depressive symptoms	.026	.011	[.007, .051]	.012	.04

Note. BC CI = bias-corrected confidence interval. Indirect effects are interpreted as cross-sectional statistical associations, not causal mediation. The paternal rejection - teacher rejection - depressive symptoms effect was estimated in a separate indirect-effect specification (teacher rejection - symptoms: b = .186, β = .13), whereas Figure 4 displays the reduced-model direct path (β = .18).

Supplementary Table S3. Equality-constraint tests for principal structural paths.

Constrained path	$\Delta\chi^2(1)$	p	ΔCFI	Interpretation
Maternal rejection - Recovery	5.84	.016	−.012	Different
Maternal rejection - Impairment	6.95	.008	−.015	Different
Paternal rejection - Sensitivity	3.21	.073	−.006	Not confirmed
Paternal rejection - Recovery	4.42	.035	−.009	Inconclusive
Teacher rejection - depressive symptoms	5.31	.021	−.011	Different
Impairment - depressive symptoms	2.10	.147	−.004	Not confirmed
Paternal rejection - depressive symptoms	1.88	.170	−.003	Not confirmed

Note. A between-group difference was concluded only when $p < .05$ and $|\Delta CFI| \geq .010$.

Supplementary Table S4. TEACH threshold by KEDASY documentation (N = 465).

TEACH threshold	KEDASY documented	No KEDASY documentation	Total
Positive	75	66	141
Negative	19	305	324
Total	94	371	465

Note. KEDASY documentation is a pragmatic comparator, not an independent clinical gold standard. Sensitivity, specificity, and predictive values describe the TEACH threshold rule, not the final combined SEN classification.

Supplementary Table S5. Free paths, covariances, variances, and fixed variances in the reduced seven-variable group-specific models.

Type	Without SEN	Combined SEN
ON	Maternal - Recovery	Paternal - Sensitivity
ON	Maternal - Impairment	Paternal - Recovery
ON	Maternal - Teacher rejection	Paternal - Impairment
ON	Paternal - Depressive symptoms	Paternal - Teacher rejection
ON	Recovery - Depressive symptoms	Sensitivity - Depressive symptoms
ON	Impairment - Depressive symptoms	Recovery - Depressive symptoms
ON	—	Teacher rejection - Depressive symptoms
ON	—	Impairment - Depressive symptoms
WITH	Maternal WITH paternal rejection	Maternal WITH paternal rejection
WITH	Sensitivity WITH Recovery	Sensitivity WITH Recovery
WITH	Sensitivity WITH Impairment	Sensitivity WITH Impairment
WITH	Recovery WITH Impairment	Recovery WITH Impairment
Free variance	Sensitivity	Sensitivity
Free variance	Recovery	Recovery
Free variance	Impairment	Impairment
Free variance	Depressive symptoms	Depressive symptoms
Fixed variance	Maternal rejection	Maternal rejection
Fixed variance	Paternal rejection	Paternal rejection
Fixed residual variance	Teacher rejection	Teacher rejection
Total free parameters	14	16

Note. ON denotes a structural regression path; WITH denotes an estimated covariance. Four variances are free in each group: the residual variances of Sensitivity, Recovery, Impairment, and depressive symptoms. Maternal and paternal rejection variances and the teacher-rejection residual variance are fixed as specified in the model inputs. The parental covariance and three covariances among emotional-reactivity components are free. Other candidate ON paths and residual covariances are fixed at zero in these reduced fits. The separate expanded multigroup comparison specification for Table S3 is not counted here. The ON entries in the two group columns are independent lists; dashes are alignment placeholders where one group has fewer free paths, not constraints on the path displayed opposite.

Supplementary Table S6. Correlation-derived three-predictor checks for emotional reactivity.

Group	Outcome	Maternal β	Paternal β	Teacher β	Implied R^2
Without SEN	Sensitivity	.072	.122	−.045	.027
Without SEN	Recovery	.195	.067	−.017	.054
Without SEN	Impairment	.061	.135	.064	.041
Combined SEN	Sensitivity	.270	−.093	.047	.061
Combined SEN	Recovery	.180	.068	.019	.051
Combined SEN	Impairment	.246	.027	.079	.082

Note. Standardized coefficients were calculated as $R_{xx}^{-1}r_{xy}$ using the rounded zero-order correlations in Tables 1 and 2, computed with available observations for each pair; predictor order is maternal, paternal, and teacher rejection. Because the reduced Mplus models used FIML, these descriptive pairwise matrices are not their analysis covariance matrices, and the derived values should not be treated as fitted path coefficients. R^2 is the corresponding matrix-implied value. For the without-SEN Impairment outcome, a separate age-expanded analysis additionally estimated age $\beta = .221$; with age approximately uncorrelated with the three rejection predictors, its incremental R^2 was .049 and the reported expanded-model R^2 was .090, compared with .041 implied by the three-predictor correlation matrix. In the combined-SEN group, separate equations adding age to the three rejection predictors yielded age $\beta = .136$ for Sensitivity, .096 for Recovery, and .261 for Impairment. The associated incremental R^2 values were .01854, .00915, and .06823, respectively, yielding expanded-equation R^2 values of .080, .060, and .150. Age was approximately uncorrelated with the three rejection predictors in these calculations. These correlation-based calculations are separate from the reduced path models in Figures 3–4 and their reported fit statistics.

Supplementary Table S7. Complete coefficients for freely estimated structural paths in the reduced group-specific models.

Group	Outcome	Predictor	b	SE	β	p
Without SEN	Recovery	Maternal rejection	.024	.011	.07	.029
Without SEN	Impairment	Maternal rejection	.080	.030	.10	.008
Without SEN	Teacher rejection	Maternal rejection	.164	.041	.26	<.001
Without SEN	Depressive symptoms	Paternal rejection	.105	.049	.15	.031
Without SEN	Depressive symptoms	Recovery	.333	.125	.16	.008
Without SEN	Depressive symptoms	Impairment	.200	.051	.22	<.001
Combined SEN	Sensitivity	Paternal rejection	.0245	.0400	.049	.540
Combined SEN	Recovery	Paternal rejection	.016	.008	.06	.041
Combined SEN	Impairment	Paternal rejection	.105	.043	.16	.014
Combined SEN	Teacher rejection	Paternal rejection	.140	.038	.33	<.001
Combined SEN	Depressive symptoms	Sensitivity	.092	.067	.08	.170
Combined SEN	Depressive symptoms	Recovery	.570	.315	.25	.070
Combined SEN	Depressive symptoms	Teacher rejection	.256	.113	.18	.023
Combined SEN	Depressive symptoms	Impairment	.352	.090	.38	<.001

Note. b = unstandardized path coefficient; SE = standard error; β = STDYX-standardized path coefficient. All freely estimated ON paths in the two reduced seven-variable models are listed, including nonsignificant paths. The reduced combined-SEN paternal rejection - Sensitivity estimate is b = .0245 (SE = .0400, β = .049, p = .540). The b = .13 and β = .26 estimates refer to the separate indirect-effect specification used in Table S2 and Figure 4, and should not be substituted for this reduced-model coefficient. Table S5 identifies paths fixed at zero, four freely estimated covariances and four free variances, and the three fixed variances. The coefficients here refer to the FIML reduced group-specific fits, not the separate pairwise correlation-derived calculations in Table S6; zero-order correlations in Tables 1 and 2 are descriptive and are not estimates of the reduced model's corresponding standardized ON paths. Depressive-symptom R^2 values of .28 (without SEN) and .31 (combined SEN) reported in the Results derive from separate expanded outcome specifications, not from the reduced models listed here.

Supplementary Table S8. Expanded depressive-symptom equations with age and sex covariates.

Group	Predictor	β
Without SEN	Paternal rejection	.150
Without SEN	Recovery	.160
Without SEN	Impairment	.220
Without SEN	Age	.221
Without SEN	Sex (male = 1)	.284445
Combined SEN	Sensitivity	.064794
Combined SEN	Recovery	.202481
Combined SEN	Impairment	.307771
Combined SEN	Teacher rejection	.145786
Combined SEN	Age	.100
Combined SEN	Sex (male = 1)	.100

Note. Sex was coded male = 1, female = 0. Standardized β values and R^2 come from the separate expanded depressive-symptom equations, not the reduced Figure 3–4 fit statistics or Table S7. Without SEN $R^2 = .280$; combined SEN $R^2 = .310$. Coefficients are reported to their original output precision.
